# JAK-STAT3信号通路与非小细胞肺癌生物学行为相关性的研究进展

**DOI:** 10.3779/j.issn.1009-3419.2010.02.16

**Published:** 2010-02-20

**Affiliations:** 250021 济南，山东大学附属省立医院胸外科 Department of Thoracic Surgery, Provincial Hospital Affiliated to Shandong University, Jinan 250021, China

在世界上许多国家肺癌都是肿瘤致死的首要因素。于是针对肺癌生物学行为的研究也迅速开展起来, 其中JAK-STAT3信号通路与非小细胞肺癌(non-small cell lung cancer, NSCLC)生物学行为相关性的研究逐渐受到重视。越来越多的证据表明, 肿瘤的发生是基因改变累积的结果。JAK-STAT3信号传导通路在大量人类肿瘤中被激活, 参与肿瘤的发生、发展, 成为目前肿瘤信号传导通路研究的热点。本文就此信号通路与NSCLC生物学行为相关性的研究进展方面进行简要综述。

## 1 JAK-STAT3信号通路的研究现状

### 1.1 STAT3分子的结构和功能

STAT3蛋白大约由770个氨基酸组成, 有三种异构体:STAT3α(即一般意义所指的STAT3)、STAT3β和STAT3γ。STATs分子结构相似, 被分成与功能相关的几个结构域, 从氨基端到羧基端依次为:氨基末端结构域、coiled-coil结构域、DNA结合结构域、linker结构域、SH2结构域(Src-homology-2 domain)、羧基末端转录活化结构域(transcriptional activation domain, TAD)^[1]^。Caldenhoven等^[2]^从嗜酸性粒细胞cDNA文库中分离并克隆了STAT3异构体STAT3β, 同STAT3α相比, STAT3β在羧基端第2145位-第2195位少50个碱基对, 导致其蛋白末端55个氨基酸残基被7个独特的氨基酸残基所替代。STAT3γ是STAT3α的限制性蛋白水解产物, 它不具有STAT3α末端的TAD, 是STAT3α的一种显性阴性异构体。

STAT3在早期胚胎发育和许多正常组织分化中发挥着不可缺少的重要作用^[3-5]^。它广泛存在于不同类型的细胞和组织中, 在大脑、心脏、肝脏、睾丸和胸腺等组织中含量较高, 在脾脏中也可检出。此外, STAT3还参与细胞生长、分化、恶性转化及凋亡抑制等活动的调控^[6]^。

### 1.2 JAK-STAT信号通路的概念及生理作用

JAK蛋白酪氨酸激酶(Janus protein tyrosine kinase)是近些年来鉴定出来的在细胞因子信号传递过程中起重要作用的蛋白酪氨酸激酶。它可在细胞因子受体与相应配基结合后活化, 并进而激活另一种信号蛋白分子“信号转导子和转录激活子”(signal transducer and activator of transcription, STATs), 从而起到诱导目的基因表达的作用。JAK蛋白酪氨酸激酶家族共包括4个成员:JAK1、JAK2、JAK3和TYK2。而STATs家族成员则有STAT1、STAT2、STAT3、STAT4、STAT5a、STAT5b和STAT6, 分别由不同的基因编码。STATs被JAK磷酸化后发生二聚化, 然后穿过核膜进入核内调节相关基因的表达, 这条信号通路称为JAK-STAT信号通路。STATs蛋白广泛表达于机体不同类型的细胞和组织中, 参与细胞生长、分化、凋亡等多种生理功能的调控, 并与炎症、肿瘤和免疫反应关系密切^[7]^。

### 1.3 JAK-STAT信号通路的激活及调节

#### 1.3.1 JAK-STAT信号通路的激活

目前研究显示, 多种细胞外蛋白可与细胞膜表面的特异受体结合, 通过酪氨酸激酶(常为JAK家族成员, 包括JAK1、JAK2、JAK3和TYK2, 有时是Src家族成员)使受体酪氨酸磷酸化而活化, 进而激活胞浆中的STAT3分子, 这些STAT单体即形成同源或异源二聚体而作为转录因子进入细胞核调控靶基因转录。常见的细胞外信号蛋白有3类:细胞因子(cytokine):IL-6等白介素、IFNα/β等; 受体酪氨酸激酶(receptor tyrosine kinases):EGF、PDGF、IGF等; 非受体酪氨酸激酶(non-receptor tyrosine kinase):Src、Abl等。

与细胞因子受体不同的是, 生长因子类受体(如EGFR等)本身即具有内在的酪氨酸激酶活性, 与其配体结合而活化后可直接磷酸化STAT3分子。而非受体酪氨酸激酶则可直接引起STAT3酪氨酸磷酸化而激活此信号传导通路。上述几种激活途径间还存在一些协同作用, 如PDGF可通过Src激活STAT3;而Src和JAK可协同介导STAT3持续活化。

#### 1.3.2 JAK-STAT信号通路的调节

在细胞胞浆及胞核中存在许多STAT反应蛋白, 从STAT分子的激活直至启动靶基因转录等诸多环节中与STAT分子相互作用而对信号传导过程进行调节。

##### 1.3.2.1 正性调节

STAT3分子翻译后的修饰极大影响其转录活性。STAT3分子第727位丝氨酸残基发生磷酸化可使其获得最大的转录活性^[8]^。另外在受体水平也存在正性调节机制, 当配体和其相应受体相结合并使其活化后, 其邻近的受体可通过交互磷酸化或使非特异抑制因子的作用下调, 而使通路得以加强。

##### 1.3.2.2 负性调节

在信号通路发挥作用的整个过程中还存在着诸多的负性调节因素, 如SOCS-1(suppressor of cytokine signaling-1)、PIAS(protein inhibitor of activated STAT)、胞浆内蛋白酪氨酸磷酸化酶(protein tyrosine phosphatases, PTP)、GRIM-19(genes associated with retinoid-IFN-induced mortality)等, 均可通过不同机制负性调节该通路。

##### 1.3.2.3 其它信号传导通路与STAT3信号传导通路的交互作用

细胞内信号的传递十分复杂, 不只是依靠单一的信号传导通路完成, 往往还需要多条信号传导通路的协同作用调节信号传导。例如:MAPK信号传导通路即可通过其家族成员ERK2、JNK1及p38等, 磷酸化STAT3分子羧基末端结构域中的有关序列, 而影响STAT3的酪氨酸磷酸化和DNA结合, 抑制其转录活性。而阻断此通路则可逆转这种抑制, 从另一角度证明了该通路对STAT3通路的抑制作用。

STAT3和其它信号传导通路通过交互作用共同调节基因的表达, 对准确调节细胞生物功能是十分重要的。进一步了解各信号传导通路之间的交互作用可以更精确的分析细胞对不同外界刺激所作出的复杂反应。

### 1.4 JAK-STAT3信号通路在肿瘤发生发展中的作用

近来的众多研究也显示, JAK-STAT通路的异常与多种肿瘤的发生密切相关。信号转导子及转录激活子3(signal transducer and activator of transcription 3, STAT3)与多种肿瘤的增殖分化、细胞凋亡、免疫逃避、血管新生、侵袭转移等密切相关。它在许多造血系统肿瘤及很多实体肿瘤中均有异常表达, 例如:白血病、乳腺癌、头颈部肿瘤、皮肤癌, 脑恶性胶质细胞瘤、前列腺癌、肺癌及肝癌等^[9-15]^。STAT3在细胞内起着重要的信号传递作用, 负责将细胞外的信号传递至细胞核, 通过诱导靶基因转录表达生物刺激的效应作用。STAT3的激活可以延长细胞的生存周期, 可能是因为其激活的靶基因就是*Bcl-2*、*Bcl-xl*、*Mcl-1*、*Survivin*等抗凋亡基因^[16]^。Bcl-2家族由多个促凋亡和抑凋亡蛋白组成, 其中Bcl-2、Bcl-XL、Mcl-1等抗凋亡蛋白可以通过抑制线粒体对细胞色素C的释放而抑制多种促凋亡因素诱导的细胞凋亡; 存活素(Survivin)是最小的凋亡抑制蛋白(IAPs), STAT3通路的持续活化可通过上调抗凋亡蛋白的表达来抑制肿瘤细胞发生凋亡, 与肿瘤的发生发展密切相关。另外还有增殖相关蛋白Cyclin D1和Myc, 以及促血管发生因子VEGF等也受JAK-STAT3信号通路的调控。而STAT3与c-Jun合作抑制Fas的表达, 也可能阻碍癌细胞凋亡^[17]^。此外, 还有其它的一些基因受到STAT3的间接调控, 这其中的许多基因都可能与肿瘤的发生、发展有关。而且众多研究显示, 如果抑制STAT3的表达将使肿瘤的生长受到抑制并导致肿瘤细胞的凋亡。

#### 1.4.1 JAK-STAT3信号通路与肿瘤细胞运动

肿瘤细胞的运动能力是肿瘤侵袭转移的重要因素之一, 且两者常常表现出正相关关系, 而且许多涉及细胞运动的细胞生理活动中往往存在JAK-STAT3信号通路的过度激活^[18]^。已经有研究显示, STAT3参与调控斑马鱼发育早期原肠运动中细胞的迁移^[19]^。还可以通过调节细胞外基质成分, 比如纤连蛋白等来调节细胞形态并影响细胞运动^[20]^。JAKSTAT3信号通路的过度激活也更加有利于细胞伸出突起和伪足, 而利于它们迁移^[21]^。可见JAK-STAT3信号通路在细胞的粘附、迁移等过程中都发挥着极其重要的作用。

#### 1.4.2 JAK-STAT3信号通路与肿瘤血管形成

肿瘤血管形成是指肿瘤细胞诱导的毛细血管生长以及肿瘤中血液循环建立的过程。随着肿瘤体积的逐渐增加, 单纯通过弥散获得的氧气和营养物质已不能满足肿瘤生长的需要, 如果没有新生血管长入, 肿瘤组织将保持休眠甚至发生退化^[22]^。血管内皮生长因子(vascular endothelial growth factor, VEGF)是刺激血管内皮细胞增殖、迁移、诱导血管形成的重要因子, 对恶性肿瘤的浸润和转移有重要意义^[23]^。实验^[24, 25]^证明STAT3可直接调控VEGF的转录, 因此肿瘤细胞中JAK-STAT3信号通路的持续过度激活可通过VEGF而促进肿瘤血管形成, 导致肿瘤的浸润和转移。

#### 1.4.3 JAK-STAT3信号通路与肿瘤对细胞外基质的降解

细胞外基质(extracellular matrixc, ECM)的降解是肿瘤侵袭转移的重要步骤之一。而基质金属蛋白酶就是降解ECM的重要成分。基质金属蛋白酶2(matrix metalloproteinases, MMP-2)为明胶酶的一种, 能够降解细胞外基质和基底膜的主要成份-Ⅳ型胶原, 目前已被证实MMP-2在乳腺癌、结直肠癌、胃癌等多种恶性肿瘤组织中表达和活性升高, 并且与肿瘤的转移能力密切相关^[26, 27]^。已经有众多实验证明, JAK-STAT3信号通路可能通过直接或间接调节*MMP-2*基因的表达参与肿瘤对细胞外基质的降解^[9]^。

#### 1.4.4 JAK-STAT3信号通路与肿瘤的免疫逃避

研究^[28]^显示人类机体免疫系统对肿瘤细胞的浸润通常表现出没有反应或呈耐受状态, 即出现免疫逃避(immune evasion)有些与肿瘤相关的免疫细胞也会出现STAT3的高表达并与该肿瘤发展相关。肿瘤细胞的JAK-STAT3通路持续激活可抑制机体免疫系统对肿瘤的相关应答, 从而介导免疫逃避, 如:JAK-STAT3通路持续激活可抑制肿瘤细胞产生促炎介质(pro-inflammatory medium); 肿瘤细胞的STAT3通路持续激活可抑制机体的先天性免疫反应; 肿瘤细胞的JAK-STAT3通路持续激活可抑制机体的获得性免疫反应。

由此可见, 肿瘤中持续活化的JAK-STAT3信号既可以通过增强肿瘤细胞运动能力, 促进肿瘤血管新生及对ECM进行降解, 以提高肿瘤侵袭、转移能力; 又可通过抑制炎症介质的释放、抑制机体免疫功能而促成肿瘤细胞的侵袭、转移。

## 2 JAK-STAT3信号通路在NSCLC研究领域中的研究现状

肺癌是严重威胁人民健康的疾病之一, 随着我国工业化的进展, 肺癌的发病率呈上升趋势, 现已位居恶性肿瘤发病率和死亡率之首。曾有调查显示, 肺腺癌的发病率占到肺癌发病率的近40%, 且具有早期血行转移, 对放化疗不敏感等特性。针对肺癌干细胞的研究^[29]^也提示肺癌具有高度的恶性及转移潜能。随着近年来针对JAK-STAT3信号通路与NSCLC关系的研究, 又给它的治疗带来了新的契机。

已经有实验^[30]^证明在NSCLC组织中STAT3的表达阳性率明显高于癌旁组织中的表达, 其中低分化组中STAT3表达水平高于高分化组, 有淋巴结转移组中STAT3表达水平高于无淋巴结转移组, 说明STAT3表达与NSCLC的组织分化程度及淋巴结转移有关。

Eric等^[31]^对176例早期NSCLC的研究以及近年诸多相关研究^[32-36]^发现, pSTAT3表达与活化形式的EGFR表达呈正相关, 而且在NSCLC中往往存在*EGFR*基因的变异或者过度表达, 这样的高表达将有利于肿瘤血管的形成并增加肿瘤的侵袭性, 所以pSTAT3的活跃与肿瘤的生长和侵袭力具有相关性, 两者也因此成为肺癌治疗的新靶点; 凋亡检测发现pSTAT3表达与细胞凋亡负相关, 这是由于JAK-STAT3通路的下游抗凋亡蛋白Bcl-xl、Bcl-2、Mcl-1和c-IAP2以及增殖相关蛋白Cyclin D1和Myc等过度表达, 而导致肿瘤细胞抗凋亡能力以及增殖能力增强^[13, 37, 38]^; 大约1/2的早期NSCLC细胞的生长依赖于持续活化的JAKSTAT3信号通路。这些都提示对pSTAT3阳性的NSCLC患者使用STAT3抑制剂可抑制肿瘤生长。而且已有实验证明在肺腺癌细胞系中也存在STAT3蛋白的持续高表达, 且其下游抗凋亡基因*CyclinD1*、*Bcl-2*等均表达增高, 据此推测STAT3蛋白的组成性激活或许是肿瘤细胞维持其恶性转化表型及维持其生长的一种内在特征。

近年来STAT3成为肺癌靶向治疗的新靶点, 为NSCLC的治疗提供了新的方向, 这主要包括以下几个方面:①寡核苷酸:是反义寡核苷酸(antisense oligonucle otide, AS-ON), 它含有12个-25个碱基, 能与STAT3 mRNA结合阻止STAT3的翻译。反义srl、AT3处理NSCLC细胞株A549和H358后, 细胞中STAT3的DNA结合能力完全缺失, 细胞发生凋亡。另一种是诱饵寡核苷酸(Decoy ON)。STAT3 DecoyON指一段双链寡核苷酸, 它与STAT3靶基因启动子反应元件中STAT3识别的特异性靶DNA序列相一致, 能竞争性结合活化的STAT3, 减少STAT3与特异性反应元件的结合, 从而阻断STAT3信号途径; ②显性负性蛋白:一类是野生型显性负性蛋白, 即STAT3B, 它缺乏TAD, 能结合STAT3靶DNA反应元件, 但不能促进相应基因的转录, 从而与活化的STAT3竞争结合相应的反应元件, 阻断STAT3信号转导通路。另一类是突变型显性负性蛋白。动物实验中, 向裸鼠体内肿瘤注射STAT3显性负性表达质粒可以诱导凋亡而致肿瘤消退; ③酪氨酸激酶抑制剂:针对STAT3信号途径中上游酪氨酸激酶如JAK、Src、EGFR的抑制剂已经研制成功, 部分已进入临床各期试验。AG490是JAK酪氨酸激酶选择性抑制剂, 可以阻断JAK激酶活化, 进而影响STAT激活, 从而显著抑制肿瘤细胞的生长, 诱导肿瘤细胞的凋亡; ④RNA干扰:RNA干扰(RNA interference, RNAi)是由双链RNA(double stranded RNA, dsRNA)引发的转录后基因沉默机制。RNAi具有特异性、高效性、对dsRNA长度依赖性和靶mRNA切割位点确定性等特点, RNAi技术可以针对信号通路的多个基因或者基因族的共有序列来同时抑制多个基因的表达, 从而能更有效地抑制肿瘤生长。目前RNAi技术已经在病毒感染、肿瘤和遗传性疾病中进行了实验, 取得了良好的效果^[39, 40]^。但由于目前尚没有合适的转基因技术, RNAi在临床肿瘤治疗上的应用有待于进一步研究; ⑤受体拮抗剂:研究证实, 多种细胞外信号通过细胞表面受体激活STAT3信号传导通路, 理论上应用此受体的拮抗剂就可以达到阻断该受体活化而明显抑制STAT3活化的目的; ⑥其它:STAT3通过酪氨酸磷酸化而被激活, 而蛋白磷酸酶可使活化状态的STAT3去磷酸化, 从而抑制STAT3活性, 其中SHP是STAT3信号传导通路中的重要负调节因子。SOCS可以抑制STAT3通路持续活化, PIAS可以抑制STAT3活性而对此通路产生负调节作用。野生型*p53*基因是一种抑癌基因, 可以促进细胞凋亡及诱导G_1_期停滞, 从而抑制肿瘤细胞恶性转化; 突变型*p53*基因不具有上述正常的功能, 其产物以显性负性方式阻断了正常p53蛋白的功能。

近些年人们开始使用各种技术手段阻断JAK-STAT3信号通路, 以达到研究此信号通路与肿瘤发生、发展关系的目的。但关于JAK-STAT3信号通路与NSCLC远处转移潜能的临床系统研究尚未见报道。
